# Characterization of Extracellular Vesicles from Infrapatellar Fat Pad Mesenchymal Stem/Stromal Cells Expanded Using Regulatory-Compliant Media and Inflammatory/Hormonal Priming

**DOI:** 10.3390/cells14100706

**Published:** 2025-05-13

**Authors:** Marc Philippon, Ramy Labib, Michelle Bellas Romariz Gaudie Ley, Lee D. Kaplan, Armando J. Mendez, Thomas M. Best, Dimitrios Kouroupis

**Affiliations:** 1 Department of Orthopaedics, UHealth Sports Medicine Institute, Miller School of Medicine, University of Miami, Miami, FL 33146, USA; mcp182@med.miami.edu (M.P.J.); ramylabib@med.miami.edu (R.L.); ley.michelle@miami.edu (M.B.R.G.L.); kaplan@med.miami.edu (L.D.K.); txb440@med.miami.edu (T.M.B.); 2 Diabetes Research Institute & Cell Transplant Center, Miller School of Medicine, University of Miami, Miami, FL 33136, USA; amendez2@med.miami.edu; 3 Department of Biomedical Engineering, University of Miami, Miami, FL 33146, USA

**Keywords:** mesenchymal stem/stromal cells, extracellular vesicles, oxytocin, immunomodulation, osteoarthritis, macrophage polarization, chondroprotection, regenerative medicine, inflammatory joint disease, cartilage repair

## Abstract

Osteoarthritis (OA) remains a leading cause of disability worldwide, with no disease-modifying therapies currently available for treatment. The infrapatellar fat pad (IFP) harbors mesenchymal stem/stromal cells (MSC) with potent immunomodulatory and regenerative properties, making them a promising candidate for OA treatment. A growing body of evidence suggests that the therapeutic effects of MSC are largely mediated by their extracellular vesicles (EVs), which carry bioactive cargo that modulates inflammation and tissue repair. However, optimizing MSC-derived EVs as a cell-free therapeutic approach requires an in-depth understanding of how culture conditions and inflammatory/hormonal priming influence their functional properties. In this study, IFP-MSC were expanded in regulatory-compliant human platelet lysate (HPL) and xeno-/serum-free (XFSF) media and primed with an inflammatory/fibrotic cocktail (TIC) with oxytocin (OXT) to assess the impact on their immunophenotypic profile and EV cargo. The immunophenotype confirmed that TIC+OXT-primed MSC retained key immunomodulatory surface markers, while EV characterization verified the successful isolation of CD63+/CD9+ vesicles. Pathway enrichment analysis of both HPL- and XFSF- TIC+OXT EVs cargo identified key miRNAs associated with immune regulation, tissue repair, and anabolic signaling. Functional assays revealed that TIC+OXT EVs promoted M2-like anti-inflammatory macrophage polarization and exhibited chondroprotective properties in chondrocytes/synoviocytes inflammatory osteoarthritic assay. These findings highlight the therapeutic potential of TIC+OXT-primed IFP-MSC-derived EVs as immunomodulatory and chondroprotective agents, offering a promising strategy for OA treatment through a clinically viable, cell-free approach.

## 1. Introduction

Osteoarthritis (OA) is a leading cause of disability, affecting approximately 500 million people worldwide and ranking as the fourth highest contributor to the disability burden [1,2]. Hip and knee OA alone account for 1.12% of all years lived with disability and are associated with increased mortality and cardiovascular disease [3,4,5].

Now understood as a total-joint and periarticular disorder, OA involves pathological interactions among the infrapatellar fat pad (IFP), synovium, subchondral bone, ligaments, and joint capsule [6]. The synovium and IFP form a functional unit that serves as a site of immune cell infiltration and a source of proinflammatory and cartilage-degradative mediators [7]. Under pathologic conditions, damage-associated molecular patterns (DAMPs) drive macrophage infiltration into the IFP, promoting a shift from anti-inflammatory M2 to proinflammatory M1 macrophages. This perpetuates a cytokine- and protease-mediated inflammatory cycle, accelerating cartilage degradation, pain, and fibrosis, underscoring the need for disease-modifying therapies that address both inflammation and tissue repair [8,9,10].

Influencing the microenvironment to modulate the immune system, reduce inflammation, and promote tissue repair has been the target of mesenchymal stem/stromal cell (MSC) research. Tissue-specific lineages of MSC can be isolated from various tissue types, such as bone marrow (BM) and infrapatellar fat pad (IFP) tissues. The immunomodulatory and anabolic effects of MSC have served as the basis of their use in clinical studies targeting a multitude of inflammatory-driven diseases, such as osteoarthritis, liver fibrosis, and spinal cord injury [11,12,13]. Preliminary trials utilizing freshly isolated or culture-expanded MSC derived predominantly from bone marrow (BM) and adipose (AT) tissues have exhibited superior clinical efficacy in hyaline cartilage structural, chemical, and functional characteristics compared to current alternatives such as hyaluronic acid intra-articular injections [14]. However, concerns surrounding the safety and immunogenicity of cell-based therapies and growing evidence attributing MSC therapeutic effects to paracrine signaling have led to an interest in cell-free approaches. Therefore, the focus has been shifted towards investigating the therapeutic potential of the MSC secretome and, more specifically, their secreted extracellular vehicles (EVs).

EVs serve as vehicles for cellular export products, including lipids, proteins, and RNAs (mRNAs and miRNAs), and can modulate the function of other cells at proximal or distal sites [15]. The immunomodulatory, angiogenic, and anabolic capabilities of MSC-derived EVs are well documented through cargo analysis and preclinical studies, supporting the use of MSC EVs instead of MSC in clinical trials [11,16,17,18,19,20,21]. However, optimizing MSC-derived EVs for clinical application requires a deeper understanding of how microenvironmental cues, such as inflammatory and hormonal priming, influence their cargo and therapeutic properties.

Oxytocin (OXT), a neuropeptide hormone well known for its role in parturition and social bonding, has shown potential anabolic and chondroprotective effects in musculoskeletal tissues by promoting chondrogenic differentiation and inhibiting cartilage degradation [22,23]. In our previous studies, primed IFP-MSC with both inflammatory/fibrotic (TIC) and oxytocin (OXT) cues showed the upregulation of genes associated with anti-inflammatory and regenerative functions compared to media without OXT. Additionally, macrophages co-cultured with IFP-MSC primed with TIC+OXT were found to enhance M2 anti-inflammatory polarization compared to media without OXT. In parallel, we have demonstrated that MSC expanded with regulatory-compliant media, human platelet lysate (HPL), or xeno/serum-free (XFSF) show superior immunomodulatory and anabolic effects in vitro and in vivo compared to fetal bovine serum-expanded MSC [24,25,26].

Building on this foundation, our study investigates how the TIC-OXT priming of IFP-MSC in HPL and XFSF media influences the EV cargo quality and immunomodulatory and anabolic functionality in vitro. We hypothesized that OXT enhances the immunomodulatory cargo of EVs, attenuates the macrophage pro-inflammatory phenotype, and demonstrates anabolic effects on cartilage. To test our hypothesis, we purified and characterized MSC EVs, analyzing their miRNA cargo and assessing their effects on M1 macrophages, synoviocyte inflammatory responses, and chondrocyte homeostasis in vitro.

This study represents a significant advancement in the field of MSC-based therapies by identifying a novel approach to augment the therapeutic efficacy of MSC EVs through OXT priming. By leveraging regulatory-compliant culture conditions, we address key translational challenges associated with MSC-derived products, paving the way for the development of clinically viable, cell-free therapies for OA. Our findings provide critical insights into optimizing the miRNA cargo of MSC-derived EVs and their potential to modulate immune responses and promote cartilage repair through unique and safe media culture conditions.

## 2. Materials and Methods

### 2.1. Isolation, Culture, and Expansion of IFP-MSC

Human infrapatellar fat pad-derived mesenchymal stem cells (IFP-MSC) were isolated from IFP tissue obtained from de-identified, non-arthritic patients (*n* = 3; 2 males, 1 female, 32.0 ± 11.31 years) undergoing elective knee arthroscopy at the Lennar Foundation Medical Center at the University of Miami. No organs or tissues were procured from prisoners. All procedures adhered to the guidelines and regulations established by the University of Miami Institutional Review Board (IRB). The IRB classified this study as “not human research” due to the use of discarded tissue, thereby waiving the need for informed consent and separate tissue collection approval. IFP tissue (5–10 mL) was mechanically dissected and repeatedly washed with Dulbecco’s Phosphate-Buffered Saline (DPBS; Sigma-Aldrich, St. Louis, MO, USA) before undergoing enzymatic digestion. Digestion was performed using 235 U/mL collagenase I (Worthington Industries, Columbus, OH, USA) diluted in DPBS with 1% bovine serum albumin (BSA; Sigma-Aldrich) for 2 h at 37 °C with continuous agitation. The enzymatic reaction was subsequently inactivated by adding complete culture media, followed by centrifugation and washing. The isolated cells were seeded at a density of 1 × 10⁶ cells per 175 cm^2^ flask in two different complete media: (i) DMEM + 10% human platelet lysate (HPL, PL Bioscience, Aachen, Germany) and (ii) xeno-free, serum-free (XFSF, Fujifilm Holdings, Tokyo, Japan). After 48 h, non-adherent cells were removed through gentle rinsing with DPBS, and fresh media were replenished accordingly. MSC cultures were maintained at 37 °C with 5% (*v*/*v*) CO_2_ and expanded until they reached 80% confluency (passage 0, P0). Cells were then subcultured at a 1:5 ratio until passage 3 (P3), with detachment facilitated by TrypLE Select enzyme 1X (Gibco, Thermo Fisher Scientific, St. Bend, OR, USA).

### 2.2. Inflammatory and Hormonal Priming

P3 IFP-MSC expanded in HPL and XFSF culturing conditions were subjected to inflammatory and fibrotic priming using a TIC cocktail containing 15 ng/mL TNF-α, 10 ng/mL IFN-γ, and 10 ng/mL connective tissue growth factor (CTGF) with 1 nM oxytocin (OXT) for a duration of 72 h. The concentrations of cytokines and hormone used for priming were based on previously established dose–response studies [24,27]. Non-induced and induced cells were subsequently assessed for immunophenotypic characteristics using flow cytometry.

### 2.3. Flow Cytometric Analysis

Flow cytometric analysis was conducted on P3 IFP-MSC expanded in HPL and XFSF culture conditions pre- and post-priming (*n* = 3). A total of 2.0 × 10^6^ cells were stained with Fixable Viability Dye eFluor 780 (Invitrogen) and labeled with antibodies targeting CD10, CD73, CD90, CD105 (BioLegend, San Diego, CA, USA), CD146 (Miltenyi Biotec, Auburn, CA, USA), HLA-DR (BD Biosciences, San Jose, CA, USA), CD283, and CD284 (Invitrogen), along with the respective isotype controls. Data acquisition was performed using a CytoFLEX S (Beckman Coulter, Brea, CA, USA), with 20,000 events collected per sample, and analyzed using Kaluza analysis software version 2.2 (Beckman Coulter).

### 2.4. IFP-MSC EV Isolation and Characterization

EVs were isolated from IFP-MSC-conditioned media using a stepwise ultracentrifugation protocol combined with immunomagnetic purification. Briefly, conditioned media collected from IFP-MSC expanded in HPL or XFSF with TIC+OXT were pre-cleared through sequential centrifugation at 2000× *g* for 10 min to remove cells and apoptotic bodies, followed by ultracentrifugation at 120,000× *g* for 4 h to pellet EVs [28]. The resulting EV preparations were resuspended in PBS and stored at −80 °C for downstream analyses [21]. Pre-enriched EVs were incubated with the Dynabeads^®^-based Exosomes-Human CD63 Isolation/Detection Reagent (Invitrogen, ThermoFisher Scientific, Waltham, MA, USA) and purified according to the manufacturer’s instructions for magnetic selection. CD9 (Invitrogen) expression was used to validate the EV presence in CD63^+^-gated particles via flow cytometry. The specific fluorescent labeling of 20,000 events was analyzed on a CytoFLEX S with Kaluza 2.2.1 analysis software (Beckman Coulter) [21]. Nanoparticle tracking analysis (NTA) (NanoSight NS300, Malvern, Worcestershire, UK) was performed to determine the size distribution and particle concentration. Samples were diluted 1:10 in PBS and analyzed under the following parameters: detection threshold of 5, room temperature, 30 frames per measurement, and a total measurement time of 30 s. The reported size distribution and concentration represent the mean of five individual measurements per condition.

### 2.5. miRNA Profiling of IFP-MSC EVs

miRNAs were extracted from EVs derived from IFP-MSC cultured in HPL or XFSF media using the Total Exosome RNA and Protein Isolation Kit (Thermo Fisher Scientific, St. Bend, OR, USA) following the manufacturer’s instructions [21]. A total of 1 µg of EV miRNA was utilized for first-strand cDNA synthesis with the All-in-One miRNA First-Strand cDNA Synthesis Kit (GeneCopoeia, Rockville, MD, USA). Predesigned human MSC exosome 166 miRNA qPCR arrays (GeneCopoeia) were performed using 1000 ng of cDNA per IFP-MSC sample (*n* = X) and processed with a StepOne real-time thermocycler (Applied Biosystems, LLC, Carlsbad, CA, USA). Data analysis was conducted using GeneCopoeia’s online data analysis system. Mean values were normalized to small nucleolar RNA, C/D box 48 (SNORD48), and expression levels were calculated using the 2^−ΔCt^ method. To analyze putative miRNA interactomes, a network-centric visual analytics approach was applied using miRNet (https://www.mirnet.ca/, accessed on 10 January 2025). miRNA target gene data were obtained from the miRTarBase v8.0 database, and interactome network refinement was performed with a 2.0 betweenness cutoff. Data were visualized in a topological miRNA–gene interactome network using a force atlas layout and hypergeometric test algorithm.

### 2.6. Macrophage Polarization Assay

Human monocytes (THP-1, ATCC) were differentiated into macrophages using PMA/IO (Phorbol 12-myristate 13-acetate/Ionomycin) at a 1X concentration and subsequently polarized to M1 macrophages using M1-macrophage generation medium (PromoCell, Heidelberg, Germany). Co-cultures of PMA/IO-stimulated THP-1 macrophages with IFP-MSC-derived EVs from HPL or XFSF conditions were maintained for 48 h. The macrophage polarization status was assessed using a macrophage polarization qPCR array (ScienCell, Carlsbad, CA, USA). RNA was extracted from THP-1 cultures using the RNeasy Mini Kit (Qiagen, Frederick, MD, USA) according to the manufacturer’s instructions. Total RNA (1 µg) was used for cDNA synthesis with the SuperScript™ VILO™ cDNA Synthesis Kit (Invitrogen). A pre-designed 40-gene Human Macrophage Polarization qPCR Array (GeneQuery™ Human Macrophage Polarization Markers qPCR Array Kit, ScienCell) was performed using 1000 ng of cDNA per culture and processed with a StepOne real-time thermocycler (Applied Biosystems, LLC, Carlsbad, CA, USA). Gene expression data were normalized to GAPDH, and relative expression levels were calculated using the 2^−ΔCt^ method. Results were visualized in a stacked bar plot representing M1, M2-like, and M2 polarization states as the relative fold change of PMA/IO + THP-1/IFP-MSC sEVs compared to PMA/IO + THP-1 (reference sample, 2^−ΔCt^ = X sample/X reference sample) [21].

### 2.7. Chondropellet/Synoviocyte Co-Culture Assay

Chondrogenic differentiation was induced in IFP-MSC (0.25 × 10⁶ cells per pellet) using serum-free MesenCult-ACF differentiation medium (STEMCELL Technologies Inc., Vancouver, BC, Canada) for 15 days. Following differentiation, chondropellets were harvested and subjected to transwell co-culture with synoviocytes, with and without EVs derived from IFP-MSC cultured in HPL or XFSF media. Co-cultures were maintained in synoviocyte medium supplemented with a TIC inflammatory/fibrotic cocktail (15 ng/mL TNF-α, 10 ng/mL IFN-γ, and 10 ng/mL CTGF) for 72 h. On day 3, chondropellets were collected for histological and molecular profiling analyses.

For the histological assessment, chondropellets were cryosectioned into 6 µm frozen sections and stained with hematoxylin and eosin (Sigma-Aldrich) for a structural evaluation and 1% toluidine blue (Sigma-Aldrich) to assess chondrogenic differentiation. Microscope images of cytochemically stained tissues were acquired using a x20 objective Leica DMi8 microscope with Leica X software version 5.2.0 (Leica, Deerfield, IL, USA). Histochemical staining quantitative analysis was evaluated in 4 samples per condition and 4 microscopy fields per sample with Fiji/ImageJ 2.14.0 software.

For molecular profiling, RNA was extracted from chondropellets using the RNeasy Mini Kit (Qiagen, Frederick, MD, USA) following the manufacturer’s instructions. Total RNA (1 µg) was used for reverse transcription with the SuperScript™ VILO™ cDNA Synthesis Kit (Invitrogen). A pre-designed 88-gene Human Osteoarthritis and Cartilage Repair qPCR Array (GeneQuery™ Human Osteoarthritis and Cartilage Repair qPCR Array Kit, ScienCell) was performed using 1000 ng of cDNA per culture and processed using a StepOne real-time thermocycler (Applied Biosystems, LLC, Carlsbad, CA, USA). Gene expression values were normalized to ACTB (β-actin) as the housekeeping gene, and relative expression levels were calculated using the 2^−ΔCt^ method, applying a 34-cycle cutoff. Data were represented in a stacked bar plot, comparing chondropellets treated with IFP-MSC-derived EVs from HPL or XFSF cultures against untreated chondropellets (reference sample, 2^−ΔCt^ = X sample/X reference sample). For the functional enrichment analysis, data were processed using g:Profiler (version e108_eg55_p17_9f356ae), applying the g:SCS multiple testing correction method with a significance threshold of 0.05 [21]. The colors for different evidence codes and for the log scale in the g:Profiler functional enrichment analysis are described in Table S1.

### 2.8. Statistical Analysis

The normal distribution of values was assessed using the Kolmogorov–Smirnov test. In the presence of a non-normal distribution of the data, one-way or two-way ANOVA was performed for group comparisons Where multiple comparisons were made, Šidák’s post hoc test was applied following two-way ANOVA. All statistical analyses were performed using GraphPad Prism v7.03 (GraphPad Software, San Diego, CA, USA), and statistical significance was considered at *p* < 0.05.

## 3. Results and Discussion

OXT has recently been recognized for its anabolic and chondroprotective effects in musculoskeletal tissues, promoting chondrogenic differentiation while attenuating inflammatory and fibrotic pathways [22,23]. Our previous studies demonstrated that IFP-MSC primed with TIC and OXT cues upregulate key anti-inflammatory and regenerative genes, while also enhancing macrophage M2 polarization. Additionally, MSC expanded in regulatory-compliant HPL and XFSF media have shown superior immunomodulatory and anabolic properties compared to fetal bovine serum-expanded MSC [29]. Building on these findings, this study examines how TIC+OXT priming in HPL and XFSF culture conditions influences the immunophenotypic profile of IFP-MSC and the functional cargo of their EVs.

### 3.1. IFP-MSC Immunophenotyping and IFP-MSC EV Characterization with TIC+OXT Priming

To enable the clinical translation of primed EV therapies for OA, it is critical to establish quality control standards for both donor cells and EV products. Donor MSC should be screened for consistent responsiveness to priming cues, stable immunophenotypic profiles, and high EV productivity across passages. During EV manufacturing, batch-to-batch variability should be minimized by standardizing the culture duration, priming conditions, and EV isolation protocols. Key EV-release criteria may include nanoparticle tracking analysis, tetraspanin expression, and RNA cargo consistency. These considerations align with recent frameworks for therapeutic EV reporting and translation, including MISEV guidelines and EV-TRACK recommendations [30]. In particular, recent efforts to standardize engineered EV products emphasize the need for well-defined cell sources, cargo reproducibility, and product safety to support regulatory approval and scalability.

**Figure 1:**
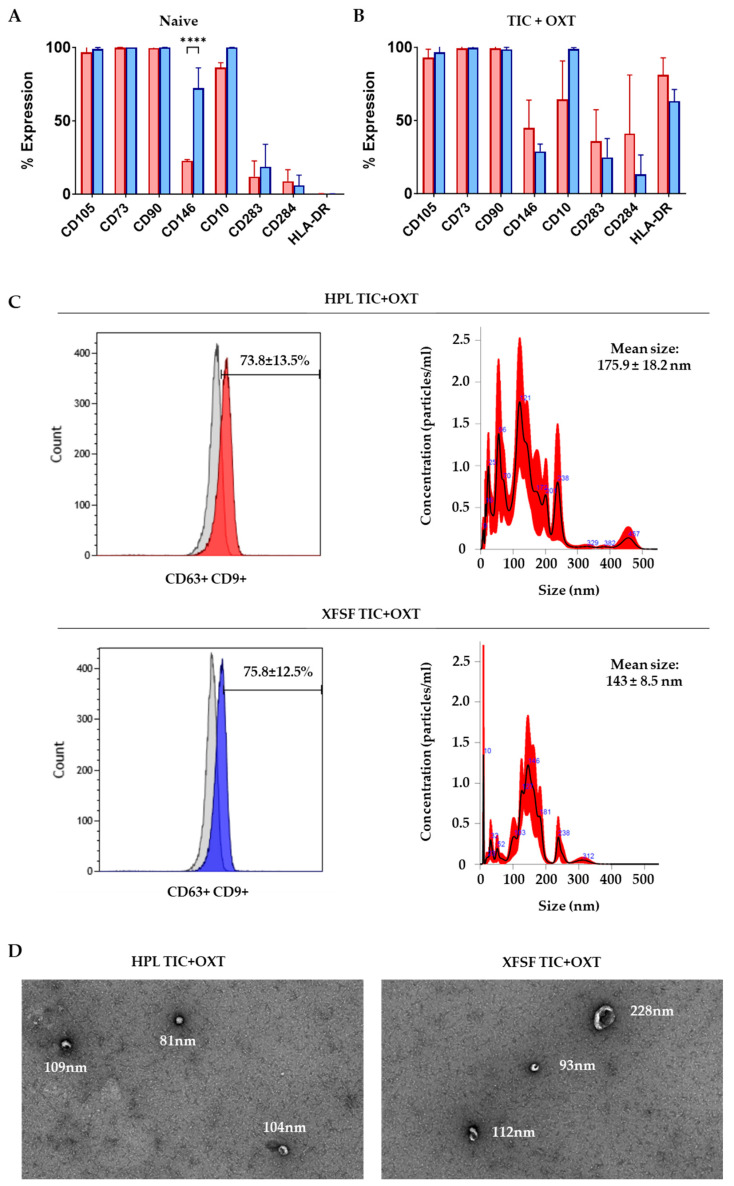
Profiling of IFP-MSC and IFP-MSC EVs expanded in HPL and XFSF media with TIC+OXT priming. (**A**) Non-primed IFP-MSC demonstrated high expression (>90%) of the MSC-defining markers CD105, CD73, and CD90 in both culture conditions. CD146 and CD10, markers associated with immunomodulation and analgesic properties, were also enriched in both HPL and XFSF conditions. **** *p* < 0.0001 (**B**) Following 72 h TIC+OXT priming, CD105, CD73, and CD90 remained highly expressed (>90%) across both conditions, while CD146 and CD10 expression increased in HPL-expanded IFP-MSC (8.68% and 4.74%, respectively) but decreased in XFSF-expanded IFP-MSC (25.44% and 1.14%, respectively). The CD283/CD284 ratio remained >1 in all conditions, consistent with a predominant TLR3-driven immunomodulatory profile. HLA-DR expression was markedly elevated in both conditions upon TIC+OXT priming, a response that has been previously reported as transient following exposure to inflammatory stimuli. (**C**) Flow cytometry analysis demonstrated that 73.8% ± 13.5% of HPL TIC+OXT EVs and 75.8% ± 12.5% of XFSF TIC+OXT EVs were CD63+/CD9+, confirming their identity as EVs. NTA revealed size distributions consistent with exosome-like EVs, with a mean particle diameter of 175.9 ± 18.2 nm for HPL-derived EVs and 143.8 ± 8.5 nm for XFSF-derived EVs. Data are presented as the mean ± standard deviation, with significance indicated by *p* < 0.05. HPL: red, XFSF: blue. (**D**) Transmission electron microscopy reveals spherical, membrane-bound vesicles with intact morphology in both HPL TIC+OXT EVs and XFSF TIC+OXT EVs. The EVs appear well-dispersed, with minimal aggregation, and are visualized using negative staining.

To assess the immunophenotypic profile of IFP-MSC expanded in HPL or XFSF media, we analyzed the expression of MSC-defining and immunomodulatory markers before and after TIC+OXT priming. Non-primed IFP-MSC exhibited high expression (>90%) of CD105, CD73, and CD90, hallmark markers of the MSC phenotype, across both conditions. Additionally, CD146 and CD10 were enriched in both culture conditions, consistent with their established roles in MSC-mediated immunomodulation and regenerative functions [31]. Notably, CD146 was significantly enriched in XFSF-expanded IFP-MSC (72.14% ± 13.88%) compared to HPL-expanded IFP-MSC (22.71% ± 0.78%, *p* = 0.0265), whereas CD10 was highly expressed in both conditions (99.91% ± 0.03% in XFSF and 86.17% ± 3.37% in HPL) but not significantly different between conditions (Figure 1A).

Following 72 h TIC+OXT priming, IFP-MSC retained high expression (>90%) of CD105, CD73, and CD90, demonstrating the preservation of core MSC characteristics despite inflammatory stimulation. However, CD146 and CD10 expression exhibited differential responses depending on the culture medium. In HPL-expanded IFP-MSC, CD146 levels increased from 22.71% ± 0.78% to 45.01% ± 18.91%, while CD10 levels decreased from 86.17% ± 3.37% to 64.41% ± 26.20%. In contrast, in XFSF-expanded IFP-MSC, CD146 levels decreased from 72.14% ± 13.88% to 28.88% ± 5.00% and CD10 remained highly expressed at 98.76% ± 0.97% (Figure 1B). CD10, a neutral endopeptidase, plays a key role in degrading substance P, a neuropeptide involved in nociceptive signaling and inflammatory joint disease progression [32,33]. Substance P secretion is increased in synovial fluid during inflammation, where it promotes fibrosis, immune cell activation, and chemokine recruitment. Our previous studies have shown that IFP-MSC and IFP-MSC EVs with higher CD10 expression exhibit greater capacity to degrade SP and suppress peripheral blood mononuclear cell proliferation, thereby mitigating synovitis and IFP fibrosis in vivo [27]. The observed reduction in CD10 expression in HPL-cultured IFP-MSC upon TIC+OXT priming suggests a differential priming response influenced by the media composition, while its consistent high expression in XFSF conditions may indicate a sustained capacity for anti-inflammatory and analgesic action.

Similarly, CD146 (MCAM) has been implicated in the pro-angiogenic and immunomodulatory functions of MSC, with previous studies linking higher CD146 expression to increased vascular support, tissue regeneration, and T cell immunosuppression [34]. On this basis, our previous data showed that the CD146 signature is correlated with innately higher MSC immunomodulatory and secretory capacities and thus therapeutic potency in vivo [31]. The increase in CD146 expression in HPL-expanded IFP-MSC upon TIC+OXT priming suggests an augmented pro-angiogenic and immunosuppressive profile, while the reduction in XFSF-expanded IFP-MSC may reflect an alternate priming response that requires further investigation.

An expression of Toll-like receptors CD283 (TLR3) and CD284 (TLR4), key mediators of MSC immunomodulation, exhibited distinct modulation between conditions. Before priming, the CD283/CD284 ratio remained >1 across both media types, a pattern consistent with the observed anti-inflammatory phenotype of IFP-MSC, characterized by elevated CD146 and CD10 expression. After TIC+OXT priming, CD283 expression increased in both conditions, rising from 18.64% ± 15.32% to 24.72% ± 12.93% in XFSF-expanded IFP-MSC and from 11.94% ± 10.72% to 35.91% ± 21.42% in HPL-expanded IFP-MSC, suggesting enhanced activation of TLR3-related immunomodulatory pathways [35]. However, CD284 expression exhibited a more substantial increase in HPL-expanded IFP-MSC, rising from 8.83% ± 7.84% to 40.92% ± 40.10%, shifting the CD283/CD284 ratio to below 1 in HPL conditions. Given that TLR4 is primarily associated with pro-inflammatory signaling, this shift may indicate a transient inflammatory response following priming, similar to the observed increase in HLA-DR expression, rather than a persistent pro-inflammatory phenotype. Finally, HLA-DR expression, which was low in non-primed cells (0.17% ± 0.09% in XFSF- and 0.19% ± 0.25% in HPL-expanded), increased significantly after priming, reaching 63.15% ± 7.97% in XFSF- and 81.08% ± 11.75% in HPL-expanded IFP-MSC. While HLA-DR upregulation is commonly associated with increased immunogenicity, previous studies have demonstrated that this response is transient following exposure to inflammatory stimuli and does not persist once inflammatory cues are removed [36]. Although some statistical comparisons between XFSF- and HPL-expanded MSC were conducted (e.g., for surface marker expression), our study was not designed to evaluate superiority between these media. Rather, our intent was to demonstrate that both regulatory-compliant media support effective MSC priming and EV generation.

The EV immunophenotypic analysis demonstrated robust CD63/CD9 double-positive EV populations, with 73.8% ± 13.5% of HPL TIC+OXT EVs and 75.8% ± 12.5% of XFSF TIC+OXT EVs expressing CD63/CD9 (Figure 1C). NTA further confirmed the successful isolation of EVs, with particle size distributions mainly within the expected range for exosome-like EVs. The mean particle diameter was 175.9 ± 18.2 nm for HPL-derived EVs and 143.8 ± 8.5 nm for XFSF-derived EVs, consistent with the size profile of MSC-derived EVs (Figure 1C).

Overall, these findings confirm that IFP-MSC exhibit an immunomodulatory phenotype, as demonstrated by the consistent expression of CD10 and CD146, markers associated with anti-inflammatory and regenerative functions. Importantly, this phenotype was preserved following TIC+OXT priming, despite modulations in the expression levels of specific surface markers, suggesting that the cells maintain their immunoregulatory properties under inflammatory and oxytocin-stimulated conditions. Additionally, EVs were successfully isolated from TIC+OXT-primed IFP-MSC, with flow cytometry confirming their CD63+/CD9+ expression and nanoparticle tracking analysis verifying size distributions consistent with exosome-like EVs. These results support the robust generation of MSC-derived EVs under both culture conditions, reinforcing their potential as key mediators of MSC-driven immunomodulation and regenerative signaling. While various post-loading strategies, such as electroporation, sonication, or chemical transfection, have been used to engineer EV cargo directly, these methods can compromise the vesicle membrane integrity, reduce yields, or introduce variability in miRNA loading [37,38,39,40]. By contrast, our priming-based approach leverages the cell’s intrinsic cargo-sorting mechanisms to enrich EVs with immunomodulatory signals in a physiologically relevant manner [41,42]. This endogenous strategy preserves EV integrity while enhancing cargo consistency, aligning more closely with scalable, regulatory-compliant EV manufacturing workflows.

### 3.2. miRNA Profiling of TIC+OXT-Primed IFP-MSC-Derived EV Cargo

**Figure 2:**
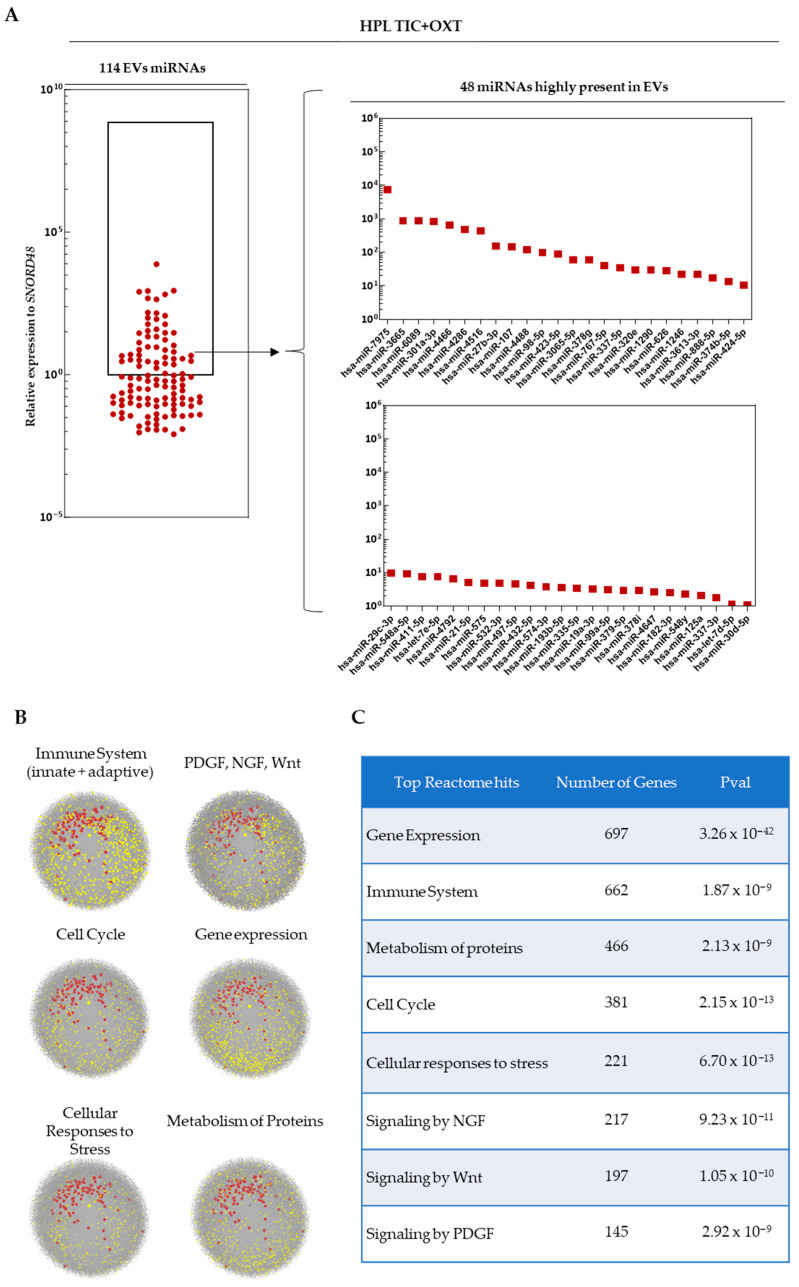
miRNA profile of EVs from TIC+OXT-primed HPL-expanded MSC. (**A**) The most highly present miRNAs included hsa-miR-7975, hsa-miR-3665, hsa-miR-6089, hsa-miR-301a-3p, and hsa-miR-4466. Additional highly present miRNAs such as hsa-miR-4286, hsa-miR-4516, and hsa-miR-27b-3p may contribute to macrophage phenotype modulation and extracellular matrix remodeling. (**B**) Pathway enrichment analysis identified major biological processes associated with these miRNAs, including immune system regulation, NGF/PDGF/Wnt signaling, protein metabolism, cell cycle control, and cellular stress responses. Grey dots: genes involved in specific biological process, red dots: detected miRNAs as EVs cargo, yellow dots: genes affected by detected miRNAs as EVs cargo. (**C**) Reactome pathway analysis of genes targeted by highly expressed miRNAs further confirms their involvement in gene expression regulation, immune system processes, protein metabolism, and key signaling pathways, such as NGF, Wnt, and PDGF, with all identified pathways showing statistically significant enrichment (*p* < 0.05).

**Figure 3:**
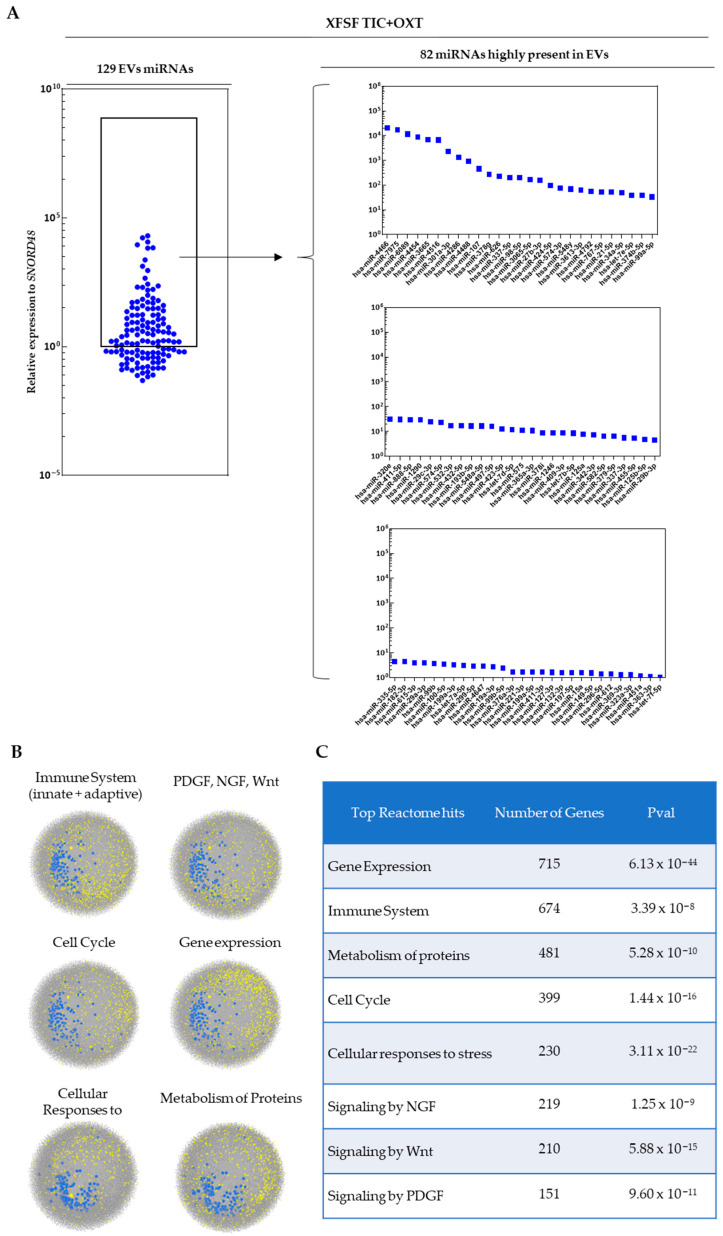
miRNA profile of EVs from TIC+OXT-primed XFSF-expanded MSC. (**A**) The most highly expressed miRNAs included hsa-miR-4466, hsa-miR-7975, and hsa-miR-6089, with the additional enrichment of hsa-miR-4454, hsa-miR-3665, and hsa-miR-4516, suggesting strong immunomodulatory potential. Notably, hsa-miR-4454 and hsa-miR-21-5p were specifically enriched in XFSF TIC+OXT EVs. (**B**) Pathway analysis revealed significant involvement in immune regulation, NGF/PDGF/Wnt signaling, extracellular matrix remodeling, and inflammatory resolution. The distinct miRNA cargo of XFSF TIC+OXT EVs suggests a potential for differential immunoregulatory effects in macrophage-driven inflammation and tissue repair. Grey dots: genes involved in specific biological process, blue dots: detected miRNAs as EV cargo, yellow dots: genes affected by detected miRNAs as EV cargo. (**C**) Reactome pathway analysis further demonstrated significant enrichment in key biological processes, with all pathways displaying statistically significant *p*-values (*p* < 0.05), reinforcing the relevance of these signaling networks in the functional impact of XFSF TIC+OXT EVs.

miRNA profiling was performed on EVs isolated from TIC+OXT-primed HPL- and XFSF-expanded MSC. A total of 114 distinct miRNAs were detected in EVs derived from HPL-cultured IFP-MSC following TIC+OXT priming, with 48 miRNAs being highly present (Figure 2A). In contrast, 129 distinct miRNAs were detected in EVs derived from XFSF-cultured IFP-MSC post-TIC+OXT priming, of which 82 were highly abundant (Figure 3A). The most highly present miRNAs in HPL TIC+OXT EVs included hsa-miR-7975, hsa-miR-3665, hsa-miR-6089, hsa-miR-301a-3p, and hsa-miR-4466. In XFSF TIC+OXT EVs, the most highly present miRNAs were hsa-miR-4466, hsa-miR-7975, hsa-miR-6089, hsa-miR-4454, and hsa-miR-3665.

Pathway enrichment analysis revealed that the miRNA cargo of TIC+OXT-primed IFP-MSC EVs was significantly associated with key biological processes related to immune regulation, cellular stress responses, signaling pathways (NGF, PDGF, Wnt), protein metabolism, and cell cycle control (Figure 2B and Figure 3B). Reactome analysis was used to determine the biological pathways most influenced by the identified miRNAs, with significant enrichment observed across multiple regulatory networks. For HPL TIC+OXT EVs, the strongest pathway association was gene expression regulation (*p* = 3.26 × 10^−42^), suggesting a widespread impact of EV cargo on transcriptional and post-transcriptional regulation in recipient cells. Other significantly enriched pathways included immune system modulation (*p* = 1.87 × 10^−9^), metabolism of proteins (*p* = 2.13 × 10^−9^), and cell cycle control (*p* = 2.15 × 10^−13^), indicating a potential role for these EVs in maintaining cellular homeostasis under inflammatory conditions. Additionally, cellular responses to stress (*p* = 6.70 × 10^−13^), NGF signaling (*p* = 9.23 × 10^−11^), Wnt signaling (*p* = 1.05 × 10^−10^), and PDGF signaling (*p* = 2.92 × 10^−9^) were significantly enriched, suggesting that the miRNA cargo may influence regenerative and reparative pathways critical for cartilage and joint homeostasis (Figure 2C).

For XFSF TIC+OXT EVs, pathway enrichment analysis similarly highlighted gene expression regulation (*p* = 6.13 × 10^−44^) and immune system processes (*p* = 3.39 × 10^−8^) as the most significantly associated pathways. Protein metabolism (*p* = 5.28 × 10^−10^) and cell cycle regulation (*p* = 1.44 × 10^−16^) were highly enriched similar to HPL TIC+OXT EVs. However, XFSF TIC+OXT EVs exhibited a stronger association with cellular responses to stress (*p* = 3.11 × 10^−22^), suggesting a possible role in cellular adaptation to inflammatory or oxidative stress conditions. Furthermore, NGF (*p* = 1.25 × 10^−9^), Wnt (*p* = 5.88 × 10^−15^), and PDGF (*p* = 9.60 × 10^−11^) signaling remained among the top pathways enriched, reinforcing the potential for these EVs to modulate tissue regeneration and immune responses (Figure 3C).

Overall, the pathway analysis suggests that TIC+OXT-primed IFP-MSC EVs carry miRNAs capable of influencing multiple biological processes relevant to inflammation, tissue repair, and cellular resilience. Although the cumulative action of the entire EV miRNA cargo likely contributes to the observed immunomodulatory effects, individual miRNAs such as miR-7975, miR-3665, and miR-6089, which are consistently abundant across both culture conditions, appear to target central regulators of inflammation, transcriptional control, and stress responses. These miRNAs may therefore act as key effectors, either independently or synergistically, in modulating the anti-inflammatory and reparative responses triggered by primed EVs. However, the combinatorial nature of miRNA-mediated regulation suggests that the synergistic activity of the miRNA repertoire plays a critical role in achieving the therapeutic effects observed.

Overall, the strong statistical significance observed in enriched pathways further supports the hypothesis that the EV cargo composition is shaped by culture conditions, with potential functional consequences for MSC-EV-based therapies.

### 3.3. TIC+OXT-Primed IFP-MSC EVs Modulate Macrophage Polarization

In OA, immune cell infiltration, particularly by monocytes and macrophages, occurs within both the IFP and synovium, contributing to a host inflammatory microenvironment [43]. These infiltrating cells interact with resident populations, promoting macrophage polarization toward a pro-inflammatory M1 phenotype, which has been implicated in OA pathogenesis [44] Notably, OA patients exhibit an increased presence of macrophages within the synovium compared to non-OA individuals, where they aggregate into multinucleated giant cell clusters. Studies have shown a significant correlation between the abundance of these multinucleated giant cells and the severity of synovitis [45]. Given this association, therapeutic approaches targeting macrophage polarization have gained interest, as emerging evidence suggests that shifting macrophages toward an anti-inflammatory M2 phenotype may help mitigate disease progression [46,47].

**Figure 4:**
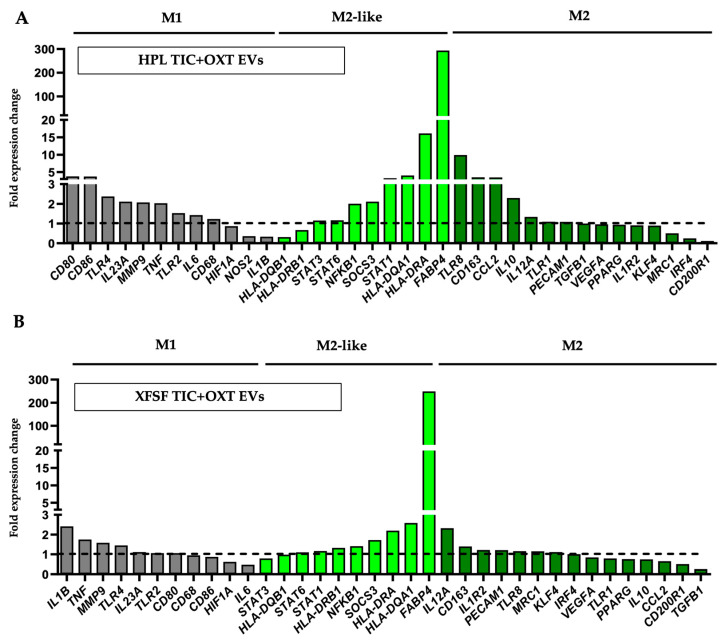
TIC+OXT-primed IFP-MSC EVs promote M2 macrophage polarization. (**A**) The co-culture of M1 pro-inflammatory macrophages with TIC+OXT-primed IFP-MSC EVs significantly promoted alternative M2 anti-inflammatory polarization, as evidenced by gene expression analysis. The exposure of M1 macrophages to HPL TIC+OXT EVs resulted in the upregulation of M2-associated markers, including FABP4, IL10, and CD163, along with a concurrent reduction in M1 markers. (**B**) The exposure of M1 macrophages to XFSF TIC+OXT EVs resulted in a more M2-like polarization effect, with robust FABP4 upregulation, indicative of a metabolic shift associated with M2 macrophage functions. All experiments (*n* = 2 biological donors) were performed independently, and data are presented as bar plots representing the mean fold expression change (grey bars: M1, light green bars: M2-like, green bars: M2 polarization, dotted line: M1 macrophages only without EVs treatment).

M1-polarized macrophages were co-cultured with EVs derived from TIC+OXT-primed IFP-MSC expanded in HPL and XFSF media, followed by the transcriptional profiling of macrophage polarization markers. Gene expression analysis revealed a shift trending towards an M2-like anti-inflammatory phenotype upon exposure to EVs. This polarization effect was observed across both conditions, but HPL TIC+OXT EVs induced a more pronounced M2-like phenotype compared to XFSF TIC+OXT EVs, putatively reflecting the observed differences in the EV miRNA cargo composition and functional potency (Figure 4).

Among the most prominently upregulated genes, FABP4 exhibited the most significant increase across both conditions, with expression levels reaching 293.48-fold in macrophages treated with HPL TIC+OXT EVs and 248.93-fold in those exposed to XFSF TIC+OXT EVs. The CD163 gene, expressing a scavenger receptor involved in tissue repair, was upregulated 3.49-fold in HPL TIC+OXT EV-treated macrophages and 1.40-fold in XFSF TIC+OXT EV-treated macrophages [48,49]. The IL10 gene, expressing a canonical M2 cytokine [50,51], was similarly elevated in HPL TIC+OXT EV-treated macrophages (2.30-fold) but showed a modest decrease in XFSF TIC+OXT EV-treated macrophages (0.74-fold). However, IL1R2, a protein that acts as a decoy receptor for pro-inflammatory IL1B to reduce inflammatory signaling, demonstrated a 1.22-fold increase in XFSF- TIC+OXT EV-treated macrophages, while being mildly downregulated in HPL TIC+OXT EV-treated macrophages (0.91-fold) [52,53,54].

Despite these trends, classic M2 markers such as PPARG (0.94-fold in HPL- and 0.76-fold in XFSF- TIC+OXT EV-treated macrophages) and MRC1 (0.51-fold in HPL- and 1.15-fold in XFSF- TIC+OXT EV-treated macrophages) were either not strongly upregulated or downregulated, suggesting an incomplete transition to a fully anti-inflammatory phenotype [55,56]. This aligns with previous studies indicating that TIC+OXT-primed IFP-MSC induce a transitional or regulatory macrophage phenotype rather than a fully polarized M2 state [29].

The expression of transitional M2-like phenotype markers, including HLA-DQA1 (4.03-fold in HPL- and 2.59-fold in XFSF- TIC+OXT EV-treated macrophages), HLA-DRA (16.13-fold in HPL- and 2.20-fold in XFSF- TIC+OXT EV-treated macrophages), and NFKB1 (2.01-fold in HPL- and 1.41-fold in XFSF- TIC+OXT EV-treated macrophages), suggests that the macrophages exhibit features of a mixed regulatory state. Importantly, prior studies have demonstrated that macrophages exhibiting intermediate levels of both M1 and M2 markers can function as immunoregulatory cells, balancing inflammatory and tissue-reparative responses [57,58,59].

Taken together, these results indicate that TIC+OXT-primed IFP-MSC EVs induce a transcriptional shift in macrophages that is consistent with an M2-like regulatory phenotype, though not a fully anti-inflammatory M2 transition. HPL TIC+OXT EVs appear to promote higher CD163 and IL10 gene expression (Figure 4A), whereas XFSF TIC+OXT EVs appear to promote a more balanced immunomodulatory profile with less pronounced inflammatory marker retention (Figure 4B). The magnitude of this effect may be influenced by multiple factors, such as the EV concentration, cargo composition, or duration of exposure. Future studies should explore whether higher EV concentrations or prolonged co-culture conditions enhance the polarization effect and further elucidate the mechanisms underlying EV-mediated immunomodulation.

### 3.4. TIC+OXT-Primed IFP-MSC EVs Modulate Chondrocyte Gene Expression and Cartilage-Associated Pathways

Synovial lining hyperplasia is a hallmark of IFP and synovium inflammation in OA. This pathological condition involves two primary synoviocyte types: type A (macrophage-like synoviocytes) and type B (fibroblast-like synoviocytes), both playing essential roles maintaining synovial homeostasis [44]. The IFP and synovium function as a combined anatomical and functional unit, serving as sites for immune cell infiltration, neovascularization, and sources of pro-inflammatory and cartilage-degrading molecules [7]. In this inflammatory microenvironment, chondrocytes respond to cytokines such as tumor necrosis factor-alpha (TNF-α) and interleukin-1 beta (IL-1β), leading to the degradation of cartilage extracellular matrix (ECM) through the action of matrix metalloproteinases (MMPs) and aggrecanases like ADAMTS-4 [14]. To replicate these conditions in vitro, we co-cultured MSC-derived chondropellets with inflamed synoviocytes, aiming to evaluate the therapeutic potential of TIC+OXT-primed IFP-MSC EVs on cartilage homeostasis.

In this study, IFP-MSC were effectively directed toward chondrogenesis using 3D micromass pellet cultures over a 15-day period. While MSC have the capacity to undergo chondrogenic differentiation, their terminal phenotype typically aligns more closely with that of cells involved in endochondral ossification rather than articular cartilage chondrocytes. However, we and others have previously demonstrated that by day 15, MSC-derived chondrocytes exhibit key chondrogenic characteristics while remaining in a pre-hypertrophic state, avoiding mineralization and terminal differentiation under in vitro conditions [25,60,61]. Subsequently, our generated chondropellets and synoviocytes were co-cultured with and without HPL- or XFSF- TIC+OXT EVs under inflammatory/fibrotic TIC conditions for 3 days.

**Figure 5:**
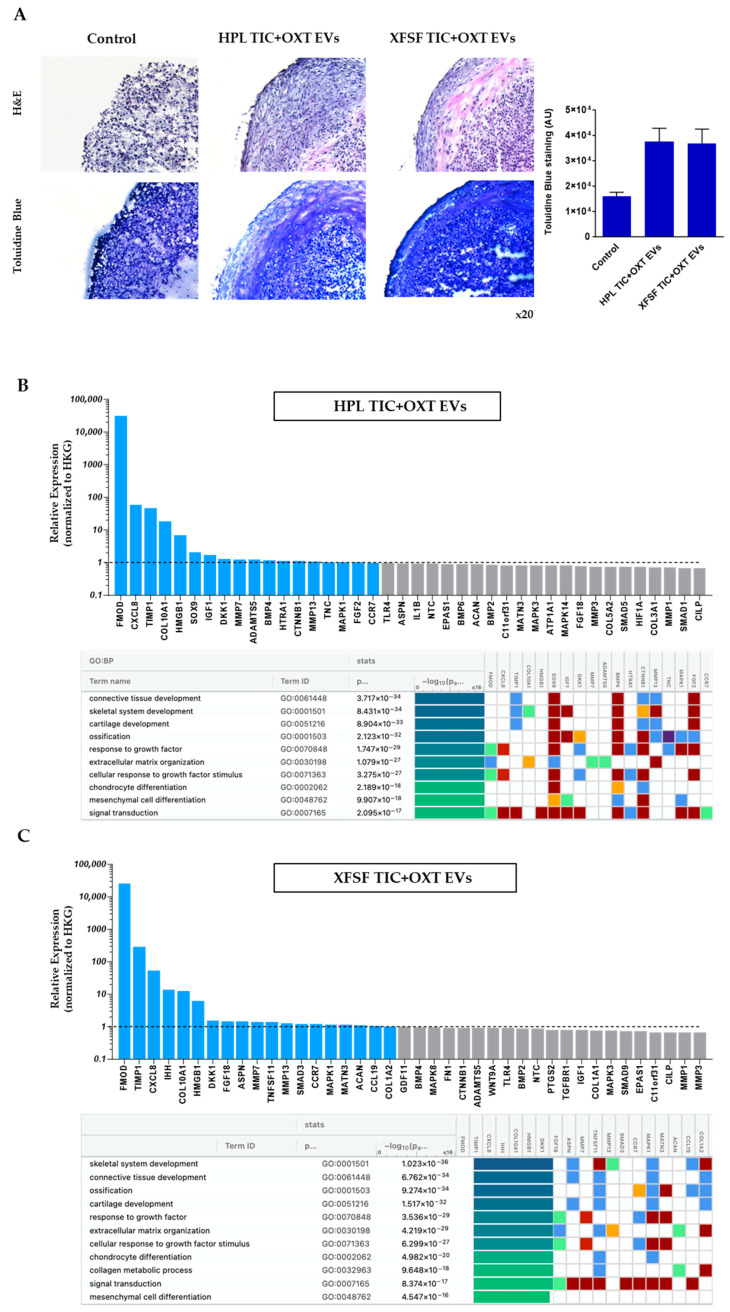
TIC+OXT-primed IFP-MSC EVs regulate chondrocyte gene expression and extracellular matrix composition. (**A**) Histological analysis of chondropellets revealed structural differences between treatment groups. Hematoxylin and Eosin (H&E) staining demonstrated increased cellular organization and matrix density in HPL- and XFSF- TIC+OXT EV-treated chondropellets compared to the control group (without EV treatment), which exhibited a loosely structured ECM and less ECM deposition. Toluidine Blue staining quantitation indicated enhanced proteoglycan deposition in both EV-treated groups, suggesting improved extracellular matrix integrity. Molecular profiling of chondropellets treated with TIC+OXT-primed IFP-MSC EVs in (**B**) HPL and (**C**) XFSF media revealed significant transcriptional changes associated with cartilage development, extracellular matrix organization, and inflammatory regulation. Bar graphs show the top 40 differentially expressed genes based on the fold-change. Both conditions resulted in the upregulation of FMOD, TIMP1, and CXCL8 genes linked to cartilage matrix remodeling and chondroprotection. A Reactome pathway enrichment analysis of highly expressed genes demonstrated their involvement in cartilage homeostasis, connective tissue development, and extracellular matrix regulation, suggesting a potential role for TIC+OXT-primed IFP-MSC EVs in supporting chondrocyte functions and tissue remodeling. Explanation of pathway analysis colors in Supplementary Table S1.

A histological analysis of chondropellets using Hematoxylin and Eosin (H&E) and Toluidine Blue staining revealed distinct differences among the non-treated and HPL- or XFSF- TIC+OXT EV groups. The non-treated group exhibited a loose structure and less cellular tissue architecture, suggesting poor matrix formation and limited cell infiltration. In contrast, the HPL TIC+OXT EV group demonstrated a denser, more organized cellular structure, indicative of enhanced cell maturation and extracellular matrix (ECM) formation. Similarly, the XFSF TIC+OXT EV group showed increased cellularity, though with some structural differences compared to the HPL TIC+OXT EV group (Figure 5A, upper panel). Toluidine Blue staining further highlighted these variations, with the non-treated group displaying weak staining and a fragmented matrix, suggesting a low proteoglycan or glycosaminoglycan (GAG) content. In comparison, both the HPL- and XFSF- TIC+OXT EV groups exhibited strong blue staining, indicating improved proteoglycan deposition and ECM integrity (Figure 5A, lower panel). These findings suggest that HPL- and XFSF- TIC+OXT EV treatments promote enhanced tissue formation and ECM deposition in vitro.

Molecular profiling revealed that in HPL TIC+OXT EV-treated chondropellets (Figure 5B), fibromodulin (FMOD), interleukin-8 (IL-8/CXCL8), and tissue inhibitor of metalloproteinases-1 (TIMP-1) were the most highly upregulated genes, with FMOD showing particularly robust expression levels. Additional genes enriched in this condition included COL10A1, SOX9, IGF1, and BMP4, which are well-established regulators of chondrocyte differentiation and cartilage matrix synthesis [62,63]. SOX9, a master transcription factor for chondrogenesis, was upregulated alongside COL10A1, a marker of hypertrophic chondrocytes, suggesting an active remodeling process [64]. Other upregulated genes included DKK1, MMP7, ADAMTS5, CTNNB1, and HTRA1, indicating involvement in Wnt signaling modulation and matrix degradation pathways [65,66]. Notably, MMP13, a matrix metalloproteinase associated with cartilage catabolism, was also elevated, highlighting the complex balance of anabolic and catabolic activity following EV treatment.

In XFSF TIC+OXT EV-treated chondropellets (Figure 5C), a similar transcriptional pattern was observed, with FMOD, TIMP1, and CXCL8 among the most significantly upregulated genes. However, IHH and COL10A1 were also highly enriched, suggesting potential differences in chondrocyte hypertrophy-related responses between the two EV treatments [67,68]. GDF11, MATN3, and ACAN genes, key regulators of cartilage homeostasis and extracellular matrix integrity, were also upregulated [69,70]. Additionally, TNFSF11, SMAD3, and CCR7 were among the differentially expressed genes, suggesting a regulatory effect on chondrocyte signaling pathways [71,72,73]. Similar to the HPL TIC+OXT EV group, the MMP13 gene was elevated, further supporting ongoing extracellular matrix remodeling processes. MMP13 and ADAMTS5 genes, both of which are involved in cartilage degradation under inflammatory conditions, along with anti-catabolic regulators such as TIMP1, suggest a potential compensatory response to matrix turnover [74,75].

Among the most highly upregulated genes in both HPL- and XFSF- TIC+OXT EV groups, FMOD, TIMP1, and CXCL8 genes exhibited robust expression changes. Specifically, FMOD, a small leucine-rich proteoglycan, is a key regulator of collagen fibrillogenesis and matrix assembly, and its upregulation suggests that EV treatment enhances cartilage structural integrity and ECM stability [76,77]. Also, TIMP1 serves as a crucial regulator of ECM turnover, preventing excessive matrix degradation—an essential function in cartilage protection against osteoarthritic changes. Finally, CXCL8 plays an integral role in chondrocyte recruitment, inflammatory signaling, and tissue remodeling, indicating that EVs may exert both protective and adaptive effects in cartilage homeostasis [76,77,78,79,80].

Pathway analysis using Gene Ontology (GO) terms was performed to identify the biological processes significantly enriched in TIC+OXT-primed IFP-MSC EV-treated chondropellets. This approach revealed strong associations with pathways involved in cartilage development, extracellular matrix organization, and skeletal system development, supporting the role of EVs in positively modulating chondrocyte functions and tissue remodeling. For HPL TIC+OXT EV-treated chondropellets, the most significantly enriched pathway was connective tissue development (*p* = 3.717 × 10^−34^), indicating a highly robust association with genes involved in tissue structural organization and repair. Other highly significant pathways included skeletal system development (*p* = 8.431 × 10^−34^), cartilage development (*p* = 8.904 × 10^−34^), and ossification (*p* = 1.231 × 10^−33^). Additionally, pathways associated with extracellular matrix organization (*p* = 1.079 × 10^−29^) and the response to growth factor signaling (*p* = 1.747 × 10^−29^) were enriched, suggesting that the EV cargo enhances anabolic and matrix-stabilizing processes in chondrocytes (Figure 5B). In XFSF TIC+OXT EV-treated chondropellets, pathway enrichment also strongly implicated skeletal system development (*p* = 1.023 × 10^−36^) and connective tissue development (*p* = 6.782 × 10^−34^) as the top biological processes. Notably, the ossification pathway (*p* = 9.274 × 10^−34^) and cartilage development pathway (*p* = 1.517 × 10^−33^) exhibited strong statistical correlations, reinforcing the role of XFSF TIC+OXT EVs in supporting cartilage structural integrity. Interestingly, XFSF TIC+OXT EVs also enriched pathways related to collagen metabolic processes (*p* = 9.648 × 10^−17^) and mesenchymal cell differentiation (*p* = 4.547 × 10^−16^), suggesting a potential effect on chondrocyte differentiation and matrix remodeling (Figure 5C).

These findings collectively demonstrate the potential of TIC+OXT-primed IFP-MSC EVs to promote extracellular matrix integrity, regulate chondrogenesis, and modulate inflammatory responses in chondrocytes. The observed transcriptional differences between HPL- and XFSF- TIC+OXT EV-treated chondropellets suggest that the functional properties of EVs may be influenced by the culture conditions of their parental MSC, potentially due to variations in the EV cargo composition. The highly significant *p*-values observed across key biological processes suggest a strong transcriptional impact of these EVs, providing mechanistic insights into their role in cartilage regeneration and tissue remodeling.

Across our experiments, results from HPL and XFSF culture conditions were largely consistent, with only minor phenotypic differences observed in parental MSC and comparable EV bioactivity across functional assays. While not a primary aim of the study, this convergence highlights the robustness of our priming strategy and suggests that it can be applied reliably across different regulatory-compliant media platforms. This is an important consideration for clinical translation, as both HPL and XFSF media are used in translational-grade manufacturing, yet differ in cost, sourcing, and regulatory classification. Our findings support the adaptability of oxytocin-based priming in diverse production settings, enhancing its potential for scalable, cell-free therapeutic development.

## 4. Conclusions

Our study demonstrates that TIC+OXT priming enhances the immunomodulatory and chondroprotective potential of IFP-MSC EVs, supporting their potential development as a cell-free therapeutic strategy for OA. IFP-MSC cultured in HPL and XFSF regulatory-compliant media retained high expression of anti-inflammatory markers (CD146, CD10) and secreted EVs enriched with immunomodulatory and anabolic miRNAs. Functionally, TIC+OXT-primed EVs promoted M2 macrophage polarization, suppressed M1 pro-inflammatory markers, and upregulated key cartilage-associated genes (FMOD, TIMP1, CXCL8), reinforcing their role in extracellular matrix remodeling and joint homeostasis. HPL TIC+OXT EVs exhibited a stronger effect on macrophage polarization, while XFSF TIC+OXT EVs contained a broader repertoire of immunoregulatory miRNAs, suggesting that both formulations hold therapeutic promise with distinct advantages. Pathway analysis confirmed their involvement in cartilage development, ECM organization, and immune regulation, highlighting TIC+OXT priming as an effective strategy to enhance MSC EV therapeutic potency. These findings establish IFP-MSC EVs as a viable, clinically translatable alternative to MSC-based therapies, offering a xeno-free, immunomodulatory, and regenerative approach for OA treatment.

## Data Availability

The original contributions presented in this study are included in the article/supplementary material. Further inquiries can be directed to the corresponding author.

## Supplementary Materials

The following supporting information can be downloaded at: https://www.mdpi.com/article/10.3390/cells14100706/s1, Table S1: The colors for different evidence codes and for the log scale in the g:Profiler functional enrichment analysis.
