# Supplementary material for: Characterization of Extracellular Vesicles from Infrapatellar Fat Pad Mesenchymal Stem/Stromal Cells Expanded Using Regulatory-Compliant Media and Inflammatory/Hormonal Priming
Source: Cells. 2025 May 13;14(10):706. doi: 10.3390/cells14100706 (PMC12109853; doi:10.3390/cells14100706)

Supplementary Table S1. The colors for different evidence codes and for log scale in the g:Profiler functional enrichment analysis.

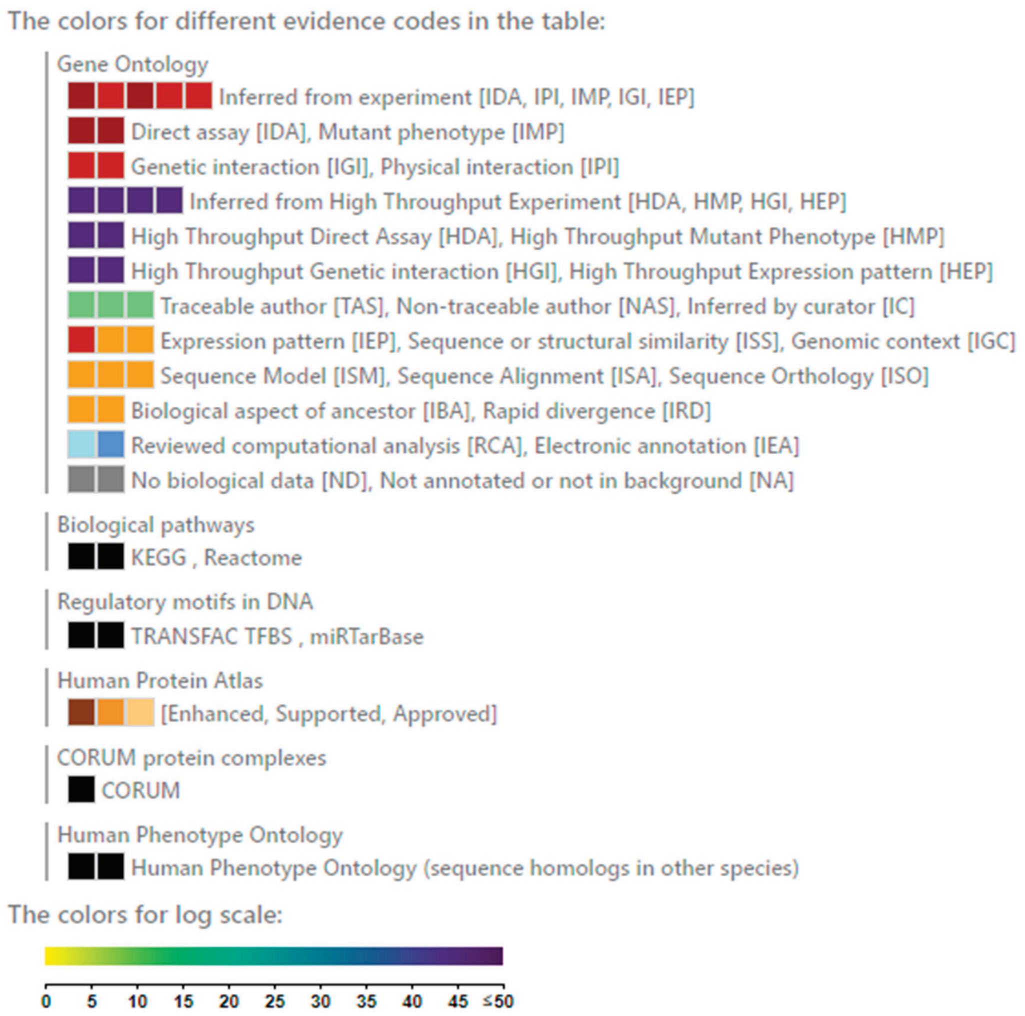

Supplement: Supplementary file 1 [file cells-14-00706-s001.zip › cells-3572469-supplementary.pdf]
